# New Technology in Percutaneous Nephrolithotomy: Application of Navigation System, Robotics, Artificial Intelligence, and Suction Access Sheath

**DOI:** 10.1111/iju.70057

**Published:** 2025-04-24

**Authors:** Kazumi Taguchi, Heiko Yang, David B. Bayne, Rei Unno, Shuzo Hamamoto, Thomas Chi, Takahiro Yasui

**Affiliations:** ^1^ Department of Nephro‐urology Nagoya City University Graduate School of Medical Sciences Nagoya Japan; ^2^ Division of Urology, Department of Surgery University of Colorado School of Medicine Aurora Colorado USA; ^3^ Department of Urology University of California San Francisco San Francisco California USA; ^4^ Department of Urology University of Alabama at Birmingham Birmingham Alabama USA

**Keywords:** artificial intelligence, innovations, percutaneous nephrolithotomy, robotic advancements, urology

## Abstract

In the dynamic field of urology, percutaneous nephrolithotomy is the gold standard procedure for treating large kidney stones and has greatly benefitted from technological advancements that improve its safety and efficacy. This review highlights significant innovations in percutaneous nephrolithotomy including integrated navigation systems, robotics, artificial intelligence, and suction access sheaths, each collectively providing greater precision and improved patient outcomes. Three‐dimensional navigation systems offer unparalleled anatomical insights, facilitate more accurate targeting, and reduce intraoperative complications, as evidenced by studies demonstrating higher success rates and shorter operation times. Robotic advancements can further refine percutaneous nephrolithotomy by offering superior control and accuracy. Emerging technologies such as the automated needle targeting with X‐ray and MONARCH platforms show the potential to reduce radiation exposure and improve procedural efficiency. Additionally, artificial intelligence in percutaneous nephrolithotomy is a novel tool for preoperative planning and outcome prediction, which can enhance decision‐making and potentially lead to customized treatment strategies. Suction access sheaths represent another important innovation that facilitates stone removal and reduces the risk of postoperative complications. Integration of these technologies can improve how urologists perform percutaneous nephrolithotomy, and it highlights the benefit of ongoing research and development in the evolving field of kidney stone surgery. Through a comprehensive analysis of peer‐reviewed studies, this manuscript delves into the advancements of the last 5 years, underscoring the transformative impact of these innovations on percutaneous nephrolithotomy and setting the stage for future developments in this critical field of urology.

## 1 Innovations in Percutaneous Nephrolithotomy

Percutaneous nephrolithotomy (PCNL) has become the preferred method for treating large kidney stones. Integrating new technologies, such as navigation systems, robotics, artificial intelligence (AI) [1], and suction access sheaths (SAS) [2], has the potential to further improve the effectiveness and safety of PCNL. These advancements have addressed the challenges associated with traditional techniques and have provided numerous benefits to surgeons and patients. Robot‐assisted PCNL, powered by AI and guided by a navigation system, allows for precise and accurate needle puncture, flattens the learning curve, and improves outcomes. SAS enables efficient stone clearance and reduces the risk of postoperative complications. These innovations have improved PCNL, making it a more efficient and effective procedure. In this study, we explored the applications of robotics, AI, navigation systems, and SAS in PCNL from recent 5‐year published peer‐reviewed studies.

## 2 Three‐Dimensional Navigation for Percutaneous Access and Stone Clearance

Three‐dimensional (3D) navigation systems are a novel innovation in PCNL (Table 1). These systems provide an in‐depth understanding of anatomy and guidance, allowing surgeons to accurately navigate and target the renal collecting system. One study found that using a 3D printing personalized percutaneous puncture guide access plate for PCNL improved the success rate of correct puncture positioning to 100% compared with the 75% success rate achieved using the standard ultrasound (US)‐guided PCNL. It also reduced the puncture time and amount of intraoperative blood loss, and these reductions were significant between the two groups [3]. Another study compared using 3D printed models to traditional PCNL‐guided access and found that the 3D printed models improved doctor–patient communication with a higher stone clearance rate (96% vs. 80%), reduced operation time (103.21 ± 13.49 min vs. 126.12 ± 25.87 min), and lower incidence of postoperative complications (6.67% vs. 22.22%) [4].

**TABLE 1:** Literatures describe the use of three‐dimensional navigation in percutaneous nephrolithotomy.

Authors	Journal	Year	Region	Study design	Patient characteristics	Case numbers	Software used	Outcome measured	Main findings
Keyu et al.	*BMC Urology*	2021	China	Randomized controlled trial	Patients who underwent PCNL	Total: 22—3D printing group: 10—control group: 12	Mimics 17.0 3‐Matic Raise 3D N2 (printer)	Success rate of single puncture	3D printing personalized PCNL guide plate showed a 100% success rate in single puncture The differences in puncture time and intraoperative blood loss were significant between the two groups (*p* < 0.05)
Huang et al.	*European Review for Medical and Pharmacological Sciences*	2021	China	Randomized controlled trial	Patients with complex kidney stones who were treated with PCNL	Total: 120—3D group: 60—control group: 60	Mimics19 3‐Matics11	Operation time, number of percutaneous punctures, blood loss, SCR, kidney injury	3D simulated puncture improved puncture accuracy, reduced the number of punctures required, decreased operation time, and lowered intraoperative blood loss significantly
Cui et al.	*Scientific Reports*	2022	China	Randomized controlled trial	Patients with complex kidney stones who met the indications for PCNL	Total: 90—3D printing group: 45—control group: 45	Mimics 17.0	Doctor–patient communication score, operation time, SCR, postoperative complications	3D printing group improved the doctor–patient communication, showed higher calculi clearance rate and lower incidence of postoperative complications
Porpiglia et al.	*European Urology*	2021	Italy	Prospective observational study with a matched pair analysis	Patients with kidney stones scheduled for ECIRS	Total: 20—3D‐MR group:10—control group: 10	Medics Blender v2.79 HoloLens (MR)	Concordance between planned puncture and intraoperative angle, puncture/treatment/radiation exposure time	3D‐MR technology demonstrated accurate needle route guidance and successful establishment of a needle access point
Wang et al.	*International Journal of Urology*	2022	China	Randomized controlled trial	Patients with renal or/and upper ureteral calculi scheduled for PCNL	Total: 61—3D‐MR group: 21—control group: 40	Mimics HoloLens2 (MR)	Coincidence rate of puncture passage, puncture time, SCR, surgical complications	3D‐MR group showed shorter puncture time, fewer puncture attempts, and higher stone clearance rate compared to the control group
Hosseini et al.	*Urology Journal*	2023	Iran	Randomized controlled trial	Patients undergoing PCNL	Total: 48—3D group: 24—control group: 24	Materialise Mimics Solidworks	Accuracy rate for lower calyceal stone access, radiation exposure time, time to stone access	3D technology in preoperative location of renal calculi significantly improved accuracy and time to access the renal calculi, while reducing radiation exposure during PCNL

Abbreviations: MR, mixed‐reality; PCNL, percutaneous nephrolithotomy; SCR, stone clearance rate.

Intraoperative simulation modules have also been used to enhance surgical outcomes in PCNL. A study showed that simulated puncture in PCNL resulted in an average intraoperative blood loss of 63.78 mL in the research group compared with 109.80 mL in the control group and a higher, but not significantly different, stone clearance rate. The incidence of complications such as penetrating kidney injury and pleural effusion was lower in the research group; however, it was not significant [5]. Another randomized controlled trial showed a mean kidney stone access time of 272 ± 109 s. Those in the intervention group who used the 3D technology had significantly shorter access and radiation exposure times than the control group. The mean size of the kidney stones was 23.06 mm, and the average surgery duration was 44.2 ± 5.6 min, with a trend toward shorter operations in the intervention group, although this was not statistically significant [6].

Furthermore, integration and real‐time navigation using mixed‐reality (MR) technologies such as holographic 3D models and augmented reality glasses have shown promising results in guiding PCNL procedures. Porpiglia et al. studied the use of 3D‐MR holograms in guiding percutaneous kidney puncture during PCNL. They found that this guidance was feasible and safe and resulted in a successful puncture of the inferior calyx in all patients. The median puncture time was 27 min with a radiation exposure of 120 s, which was less than that obtained using the standard technique. Additionally, the study indicated a higher success rate for renal puncture on the first attempt with 3D‐MR (100% vs. 50%), despite some limitations, such as small sample size and manual overlapping of hologram models [7]. Furthermore, another study observed that implementing 3D‐MR using HoloLens2 resulted in more accurate kidney positions during procedures and shorter overall puncture times with potentially better stone clearance rates than routine PCNL performed without this technology [8].

## 3 Robotic Advancements in Percutaneous Renal Access

Robotic advancements in kidney stone removal have improved PCNL. The introduction of robotics has revolutionized PCNL by improving surgical outcomes and enhancing surgeons' capabilities.

Notably, several studies have explored innovative techniques for PCNL, a surgical procedure for removing kidney stones. Li Ruipeng et al. evaluated the efficacy and safety of real‐time ultrasonography‐guided PCNL using SonixGPS navigation and demonstrated successful procedures with high stone clearance rates and no major complications [9]. Furthermore, Mazdarani et al. presented a new method for robotic‐assisted PCNL that utilized a PCNL‐guided visual algorithm to track needle movement in real time with remarkable accuracy and control frequency without requiring prior knowledge of the needle trajectory or additional position sensors [10]. Notably, 5G technology provides intraoperative tele‐assistance surgery. A prospective proof‐of‐concept study was conducted to perform PCNL using a 5G‐powered robotic‐assisted tele‐PCNL diagnostic system. There was 177 ms of delay for over 5800 km; however, 15 patients underwent successful PCNL with a 78.6% first‐renal puncture success rate. Therefore, visualizing and monitoring a PCNL probe is a challenging yet essential aspect of percutaneous renal access. Combining robotic and navigation technologies offers immediate feedback to the surgeon, improves trajectory precision, and reduces manipulation errors that occur while using the PCNL probe.

**FIGURE 1:**
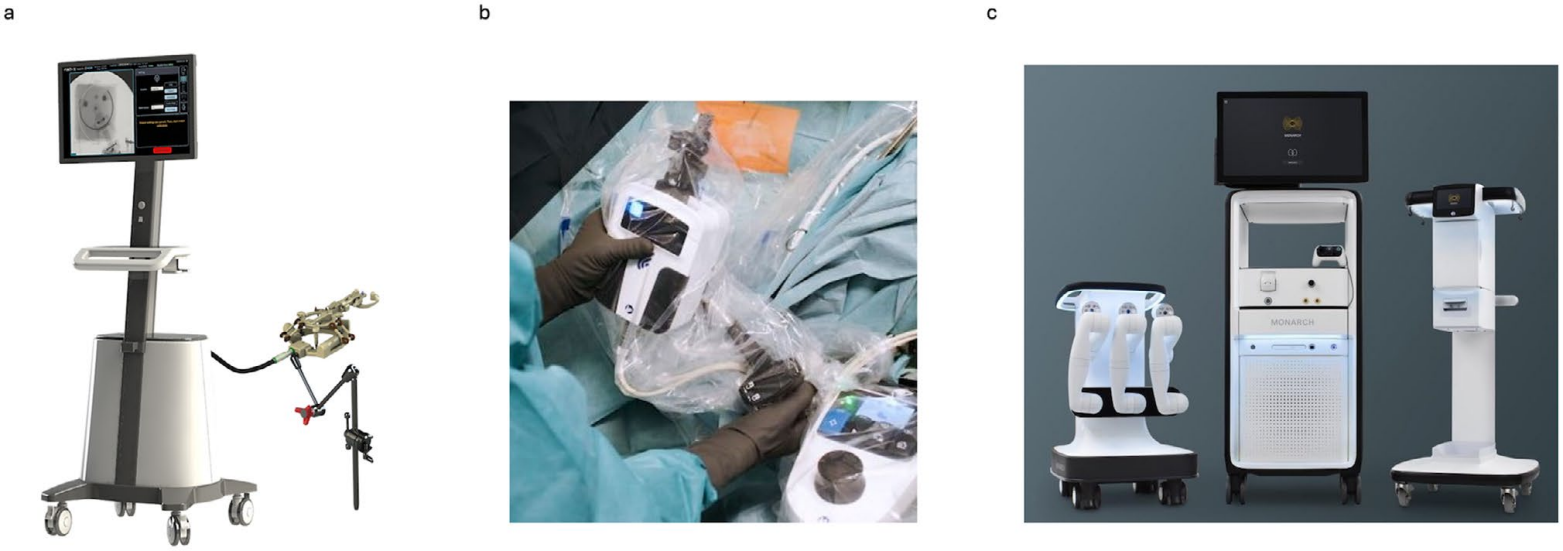
Images of FDA‐approved robotic devices for percutaneous nephrolithotomy. (a) Automate needle targeting with X‐ray. (b) Robotic platform by LARC Robotics. (c) MONARCH.

Robotic tools under fluoroscopic guidance have been more clinically applied to PCNL than PCNL‐guided puncture. Automated needle targeting using fluoroscopy (ANT‐X) (NDR Medical Technology, Singapore) provides automatic calculation and adjustment of the needle trajectory toward the targeted calyx using algorithms with AI‐trained real‐time image detection (Figure 1a) [11]. The efficacy of ANT‐X in improving percutaneous access for PCNL has been evaluated in animal and human studies, including randomized controlled trials [12]. The results showed that using ANT‐X significantly increased the success rate of puncture and reduced the puncture time compared with conventional methods, which may be beneficial to inexperienced surgeons, ultimately improving patient outcomes and expanding PCNL's adoption as a treatment for kidney stones. LARC Robotics has also released a new technology for robotic renal access and scope manipulation in PCNL (Figure 1b) [13]. Its robotic arms are controlled by joysticks, allowing precise movement and manipulation of instruments during PCNL procedures under fluoroscopic guidance.

Moreover, Lima et al. have reported a new navigation system that uses real‐time electromagnetic (EM) sensors without PCNL or fluoroscopy guidance for percutaneous kidney access [14]. The study, conducted as a proof‐of‐concept at a single academic center, involved 10 patients and demonstrated that the system enabled a successful puncture of the renal collecting system on the first attempt, with a median time to successful puncture of 20 s and no complications. Recently, the FDA approved the MONARCH platform (Ethicon Inc., Raritan, NJ, USA) for the robotic‐assisted removal of kidney stones, marking a significant advancement in PCNL use (Figure 1c). It has robotic arms and a handheld controller, enabling EM‐guided percutaneous renal puncture. In previous studies involving benchtops and cadavers, the new platform demonstrated favorable outcomes such as 80% less radiation exposure during the percutaneous access and improved ease of some tasks during the procedure [15].

The introduction of robotics, AI, and navigation systems has undeniably transformed PCNL procedures and improved surgical outcomes; however, some potential drawbacks and concerns associated with these technological advancements should be considered [16]. One primary concern is the potential for increased costs. Implementing these advanced technologies may lead to higher procedural costs, which could limit the access of patients and healthcare systems with limited resources [17]. Furthermore, a learning curve may be associated with using these new technologies, particularly for surgeons accustomed to traditional techniques. The training and education required to effectively use robotic‐assisted procedures and navigation systems may prevent widespread adoption, particularly in regions with limited access to specialized training programs.

## 4 AI in Urological Procedures

AI is a valuable tool in urological procedures, including PCNL (Table 2). A previous study described using an AI‐based decision support system to predict stone‐free status following PCNL for patients with staghorn calculi. Machine learning techniques, including dimensionality reduction and classifier models, showed that incorporating linear discriminant analysis improved classification accuracy by approximately 10% [18]. The random forest algorithm yielded the most promising results. The system demonstrated favorable accuracy in outcome prediction and could potentially assist urologists in preoperative counseling and surgical decision‐making for kidney stone removal. Al‐Azab et al. aimed to create and compare machine learning models with traditional scoring systems to predict the success of stone removal (stone‐free status) in patients undergoing PCNL. A retrospective analysis involving 320 patients showed that these machine learning models achieved higher accuracy rates, with areas under the curve (AUCs) superior to those of traditional scoring systems. Factors such as initial stone burden, length of hospital stay, and patient age significantly affected the predictive outcomes. This study also included a diverse Middle Eastern cohort and new variables such as preoperative urinary tract infection and age, which were not considered in the other scores [19]. Machine learning models were used to predict PCNL outcomes based on a comprehensive national database from the British Association of Urological Surgeons. They demonstrated moderate accuracy in predicting patient outcomes [20]. These models have been temporally validated and can be used in clinical practice for personalized risk assessment. Further external validation is needed, however.

**TABLE 2:** Literatures utilized machine learning to predict outcomes in percutaneous nephrolithotomy.

Authors	Journal	Year	Number of cases	Case characteristics	Outcome measured	Algorithms	Main findings
Hameed et al.	*Journal of Endourology*	2021	Total: 100	Patients who underwent PCNL	Accuracy in predicting the SFS	Logistic regression (LR), support vector classification (SVC), decision tree (DT), random forest (RF), K‐means clustering	81% accuracy in predicting the outcome of PCNL
Hou et al.	*Frontiers in Endocrinology*	2023	Total: 175—training set: 67—testing set: 108	Patients who underwent PCNL	SFS	Minimum absolute shrinkage and selection operator (Lasso), logistic regression, correlation filters, 10‐fold cross‐validation, transfer learning	Good discriminatory ability in both training and external validation groups with AUC values of 0.871 and 0.744
Alazab et al.	*International Journal of Nephrology and Renovascular Disease*	2023	Total: 320	Patients who underwent PCNL	SFS	Random forest (RF), support vector classifier (SVC), and extreme gradient boosting (XGB)	The accuracy of the system for predicting the stone‐free rate was found to be between 70% and 79% with an AUC between 0.751 and 0.858
Geraghty et al.	*European Urology Focus*	2024	Total: 12810—training and testing sets varied	BAUS PCNL audit	SFS and peri‐/postoperative complications	Extreme gradient boosting (XGB), deep neural network (DNN), and logistic regression (LR)	The AUC values of the outcomes after internal validation were between 0.82 and 0.94
Ibragimov et al.	*IEEE Access*	2023	Total: 248	A consecutive series of percutaneous access on a human cadaver model by MONARCH	Accuracy of needle insertions, procedure metrics, automated labeling of needle insertion activities	One‐dimensional U‐net neural network and a random forest classifier	High accuracy (94%) in automatically recognizing first needle insertions for which the Dice coefficient was 0.86 Moderate accuracy (66%) in recognizing secondary needle insertions

Abbreviations: AUC, area under the curve; BAUS, British Association of Urological Surgeons; PCNL, percutaneous nephrolithotomy; SRS, stone‐free status.

Additional investigations utilized a black‐box tool called deep learning to improve the accuracy of PCNL and enhance its utility for prediction. A previous study developed an AI‐based model using radiological characteristics, deep learning, and clinical data to predict the success of PCNL in patients with renal staghorn stones. The DTL + *R* ad signature model showed good predictive performance for stone clearance after PCNL, particularly for complex stones, such as staghorn stones. The AI‐based approach was validated with high accuracy (AUC values of 0.871 and 0.744 in the training and validation sets, respectively). The model also demonstrated high specificity in determining the likelihood of stone clearance postoperatively [21]. Another study using 18 human cadaveric models developed a deep learning framework combining a U‐net with random forests to analyze sensor data from robot‐assisted renal access procedures using the MONARCH platform. This innovative approach achieved a 94% and 66% success rate for automatically recognizing first and secondary needle insertions, respectively [22]. As other examples of deep learning modality, convolutional neural network provides accurate identification of kidney stone composition from digital photographs. Black et al. applied ResNet‐101 (Microsoft) as a multi‐class classification model to each image and achieved 71.43%–95% precision for different stone types [23].

## 5 Enhancing Kidney Stone Surgery With Suction Access Sheath

SAS is a technology that allows improved visualization and clearance of stone fragments during the procedure (Table 3). SAS reduces the frequency of instrument exchanges and can potentially decrease operating time and complications [2].

**TABLE 3:** Literatures evaluated suction access sheath impact in percutaneous nephrolithotomy.

Authors	Journal	Year	Region	Study design	Patient characteristics	Case numbers	Outcome measured	Main findings
Lievore et al.	*Journal of Endourology*	2020	Italy	Observational and retrospective study	Patients who underwent PCNL	Total: 156—smPCNL: 104—cmPCNL: 52	Operative/fluoroscopy time, patient effective dose, rate of infectious complications, and SFS	smPCNL was associated with shorter operative time, lower fluoroscopy time, lower patient effective dose, and a lower rate of infectious complications compared to cmPCNL The stone‐free rate was higher in the smPCNL group compared to the cmPCNL group (89.4% vs. 78.8%)
Lai et al.	*Journal of Endourology*	2020	China	Non‐randomized, single‐center prospective study	Patients with single renal pelvic stone	Total: 150—smPCNL: 75—cmPCNL: 75	Immediate SRS and mean perioperative renal pelvic pressure	Significant improvement in stone retrieval efficiency, reduced operative time, postoperative complications, and pain with the smPCNL Higher immediate stone‐free rate in the smPCNL group compared to the cmPCNL group (89.3% vs. 77.3%)
Gallioli et al.	*Minerva Urology and Nephrology*	2020	Italy Spain	Prospective non‐comparative study	Pediatric population (median age of 119 months)	Total: 13	SFS	The stone‐free rate was 81.3%, rising to 93.8% after ancillary procedures
Pozzi et al.	*Journal of Clinical Medicine*	2022	Italy	Retrospective observational study	Patients who underwent PCNL	Total: 287	Trifecta status (SFS without complications and no auxiliary procedures)	Trifecta status was achieved in 60% of cases after smPCNL, with stone volume and multiple calyces involved being identified as independent unfavorable risk factors
Tuoheti et al.	*Scientific Reports*	2023	China	Retrospective comparative study	Patients diagnosed with large kidney stones	Total: 132—DsmPCNL: 68—cmPCNL: 64	Primary SFS, operative time, fever rate, need for auxiliary procedures	DsmPCNL showed superior outcomes compared to cmPCNL, with shorter operative times, lower rates of auxiliary procedures, lower fever rates, and a higher primary stone‐free rate (85.3% vs. 70.3%)
Tominaga et al.	*Journal of endourology*	2023	Japan	Observational, retrospective cohort study	Patients who underwent ECIRS for staghorn stones	Total: 61 (smECIRS)	SFS and complications	The initial and final stone‐free rates were 50.8% and 91.8%, respectively, with 29.5% of postoperative fever > 38°C

Abbreviations: cmPCNL, conventional miniaturized PCNL; DsmPCNL, double suction miniaturized PCNL; PCNL, percutaneous nephrolithotomy; smECIRS, suction miniaturized endoscopic combined intrarenal surgery; smPCNL, suction miniaturized PCNL; SRS, stone‐free status.

One study compared SAS (ClearPetra, Well Lead Medical, China) with the traditional access sheath in a mini‐PCNL procedure. They found that the SAS group had shorter operative times (32.4 vs. 46.2 min), higher stone‐free rates (89.3% vs. 77.3%), lower postoperative pain levels, reduced instances of postoperative fever, and lower mean perioperative renal pelvic pressures [24]. Similarly, Lievore et al. compared two mini‐PCNL techniques: one using the MIP system (Karl Storz, Tuttlingen, Germany) with the vacuum cleaner effect and the other using SAS (ClearPetra). They found that the former technique was associated with shorter operative and fluoroscopy times and a lower rate of infectious complications than the latter. However, both methods showed similar success rates in achieving a postoperative stone‐free status [25]. The SAS is also used in pediatric cases. Groups from Italy and Spain assessed 18 mini‐PCNL procedures in children with a median cumulative stone size of 32 mm, including eight with staghorn stones. The median operative time was 128 min, and a stone‐free rate of 81.3% was achieved without any intraoperative complications or blood transfusions [26].

Another study aimed to validate the trifecta criteria, defined as stone‐free status without complication after a single session of surgery [27], in mini‐PCNLs using SAS and identify the predictors for achieving trifecta. The study found that approximately 6 of 10 patients achieved trifecta status following mini‐PCNL with SAS. The critical predictors of success included a smaller stone volume, a higher rate of single stones, shorter operative time, and using a single percutaneous tract. The chance of achieving trifecta decreases with the presence of more calyces, stone distribution across multiple calyceal groups, and larger stone volume, which are independent unfavorable risk factors for trifecta [28]. Tuoheti et al. further evaluated the effectiveness of a novel double‐sheath negative pressure mini‐PCNL using two different sizes of ClearPetra (outer: 20Fr, 13 cm; inner: 16Fr, 21 cm). The novel mini‐PCNL group showed a shorter operative time, higher primary stone‐free rate, and lower rates of auxiliary procedures and postoperative fever than the conventional mini‐PCNL group. However, there were no significant differences in the final stone‐free rate, decrease in hemoglobin levels, or stone composition between the two groups, indicating that double SAS mini‐PCNL offered certain immediate benefits but did not significantly affect long‐term outcomes associated with stone removal or blood loss [29]. Finally, SAS is applicable even in cases of endoscopic combined intrarenal surgery (ECIRS), resulting in an initial stone‐free rate of 50.8% and a final stone‐free rate of 91.8% [30]. Overall, using SAS in mini‐PCNL procedures has shown promising results, with a high stone‐free rate and lower postoperative infection rates.

## 6 The Future Direction of Technology‐Driven Percutaneous Nephrolithotomy

Future development of technology in PCNL will incorporate 3D navigation systems, robotics, AI, and SASs. These innovations can enhance the accuracy, efficiency, and safety of these procedures. Currently, the clinical and practical applications of AI for PCNL are limited to predicting postoperative stone clearance and complications, and detecting recurrence. Owing to the rising research development in surgical targeting for PCNL led by MR, AI, and robotic platforms [31], real‐time AI integration to establish surgeon support with intraoperative algorithms will be the goal of next‐generation PCNL technology. Similarly, user‐friendly 3D navigation and robotic assistance must be implemented to improve intraoperative maneuverability, precision, and visualization [32]. Combining these technologies can improve PCNL by enabling more precise stone targeting, reducing complications, and improving patient outcomes.

Regarding stone fragmentation and dusting, the suction sheath technology facilitates efficient stone clearance and may contribute to miniaturized endoscopic tool usage. The suction sheath may mitigate postoperative urinary tract infections by lowering intrarenal pressure. Additional features such as automatic irrigation with a pressure sensor or simultaneous use of a flexible ureteroscope with a pressure sensor (LithoVue Elite) during ECIRS can facilitate optimal surgical outcomes. Finally, current laser technologies, such as the thulium fiber/YAG laser [33] and high‐power holmium YAG laser with Moses technology [34], provide sufficient stone clearance, but the development of more advanced laser technologies and delivery systems may further improve the effectiveness of stone fragmentation and dusting during PCNL.

## 7 Conclusion

Advancements in PCNL technology, such as 3D navigation systems, AI integration, robotics, and SAS, have shown significant promise for improving procedural accuracy, efficiency, and safety. Using SAS has improved visualization, reduced operating times, and provided higher stone‐free rates. The integration of AI in predicting postoperative stone clearance and complications has shown high accuracy and specificity, leading to more personalized patient care. Therefore, integrating these technologies can revolutionize PCNL by enabling precise stone targeting, reducing complications, and ultimately improving patient outcomes. The future holds great promise for technology in PCNL, and there is great potential to set new standards for treatment success.

## Author Contributions

**Kazumi Taguchi:** conceptualization, data curation, formal analysis, visualization, writing – original draft. **Heiko Yang:** conceptualization, methodology, writing – review and editing. **David B. Bayne:** conceptualization, methodology, validation, writing – review and editing. **Rei Unno:** data curation, methodology, visualization, writing – original draft. **Shuzo Hamamoto:** conceptualization, investigation, resources, writing – review and editing. **Thomas Chi:** conceptualization, project administration, resources, supervision, writing – review and editing. **Takahiro Yasui:** conceptualization, conceptualization, funding acquisition, funding acquisition, project administration, project administration, resources, resources, supervision, supervision, writing – review and editing, writing – review and editing.

## Conflicts of Interest

K.T. serves as an executive at a subsidiary of NDR Medical Technology. T.Y. is an Editorial Board member of the International Journal of Urology and a coauthor of this article. To minimize bias, they were excluded from all editorial decision‐making related to the acceptance of this article for publication. The other authors declare no conflicts of interest.
